# Supplementary material for: The interactome of intact mitochondria by cross-linking mass spectrometry provides evidence for coexisting respiratory supercomplexes
Source: Mol Cell Proteomics. 2017 Dec 8;17(2):216–32. doi: 10.1074/mcp.RA117.000470 (PMC5795388; doi:10.1074/mcp.RA117.000470)
Supplement: Supplemental Data [file supp_RA117.000470_133922_0_supp_23978_fzffwf.zip › spectra_annotation/mito_spectra_annotation/471-1-12-1-8-1.pdf]

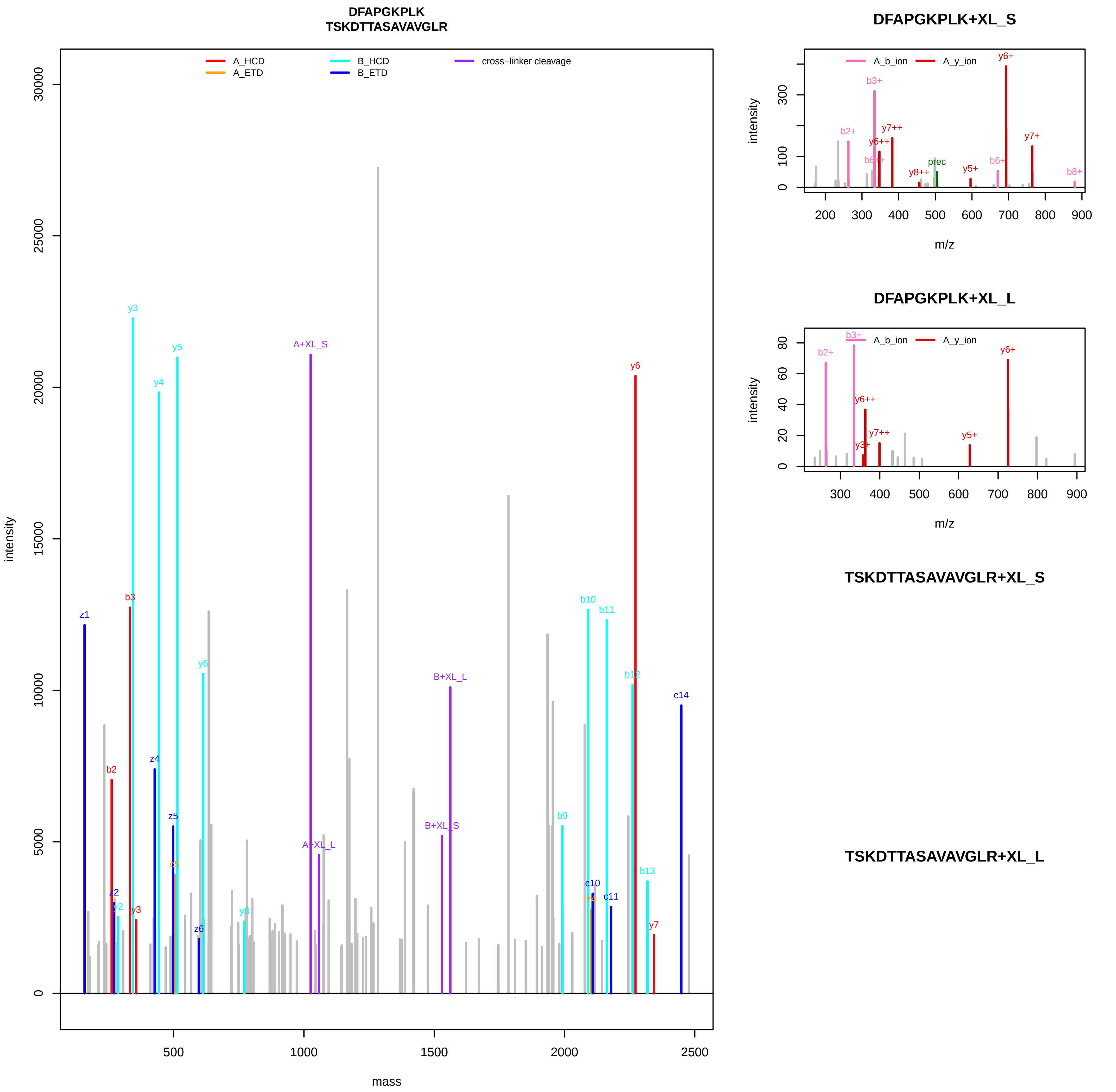
DFAPGKPLK
TSKDTTASAVAVGLR
A_HCD
A_ETD
B_HCD
B_ETD
cross−linker cleavage
intensity
mass
z1
b2
b3
y3
y4
y5
z4
z5
y6
z6
c5
A+XL_S
A+XL_L
B+XL_S
B+XL_L
b9
b10
b11
b12
b13
c10
c11
c14
y6
y7
DFAPGKPLK+XL_S
A_b_ion
A_y_ion
b2+
b3+
b6++
b6+
b8+
y5+
y6+
y6++
y7+
y7++
y8++
prec
m/z
DFAPGKPLK+XL_L
y3+
TSKDTTASAVAVGLR+XL_S
TSKDTTASAVAVGLR+XL_L
